# Supplementary figures and images for: In vitro selection of Remdesivir resistance suggests evolutionary predictability of SARS-CoV-2
Source: PLoS Pathog. 2021 Sep 17;17(9):e1009929. doi: 10.1371/journal.ppat.1009929 (PMC8496873; doi:10.1371/journal.ppat.1009929)

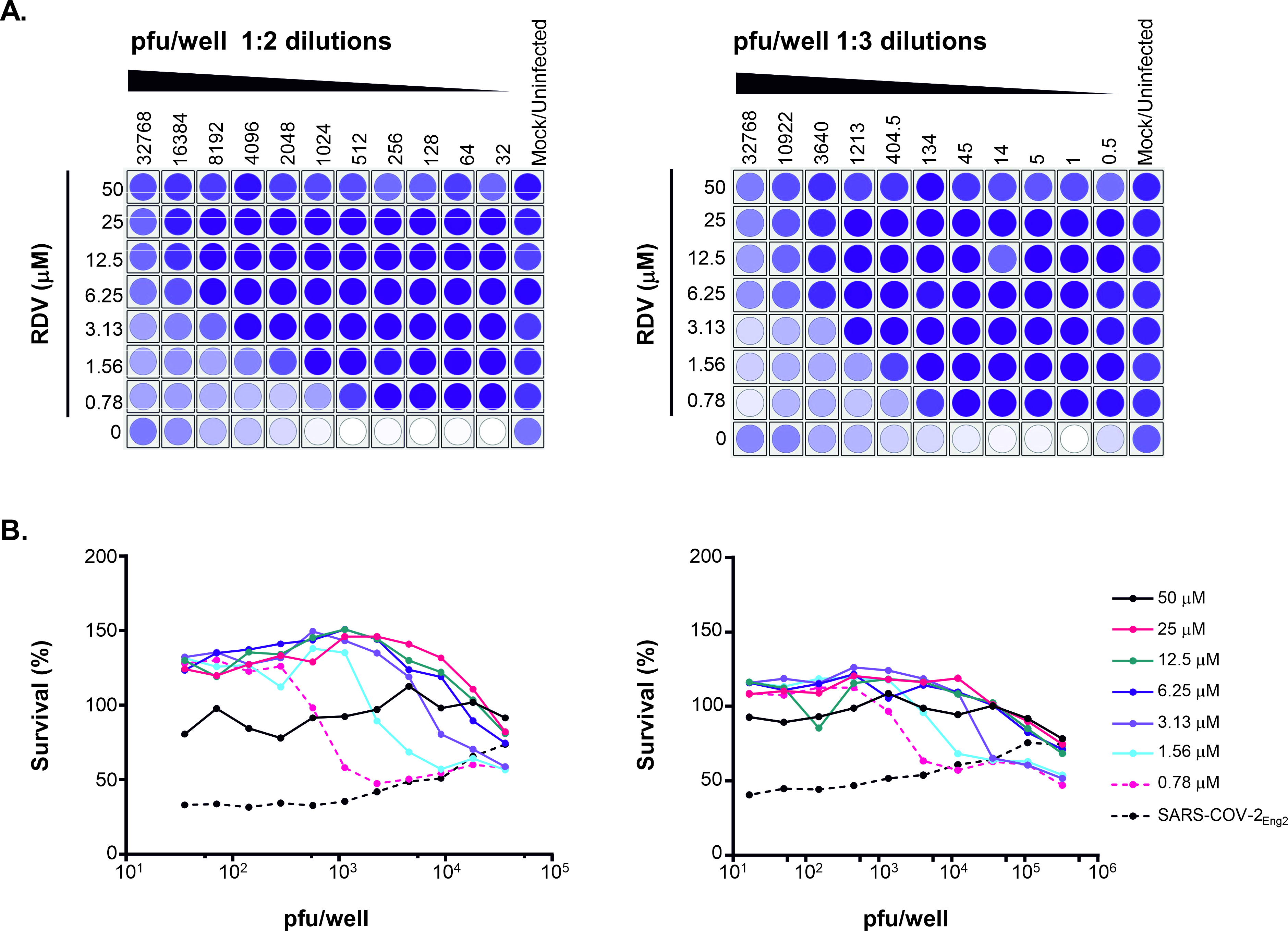

Supplement: S1 Fig — Two-fold serial dilutions of RDV from 50μM and either a two-fold (left) or three-fold (right) dilution of SARS-CoV-2. (A) Heatmap of total intensity of the stained plated generated by the Celigo. Lightest colors indicate clearance of the monolayer. (B) RDV dose dependency over a range of virus inputs. For each RDV concentration the survival (%) of the monolayer with different virus inputs per well is plotted. Values are from 1 replicate per condition. (TIF) [file ppat.1009929.s001.tif]

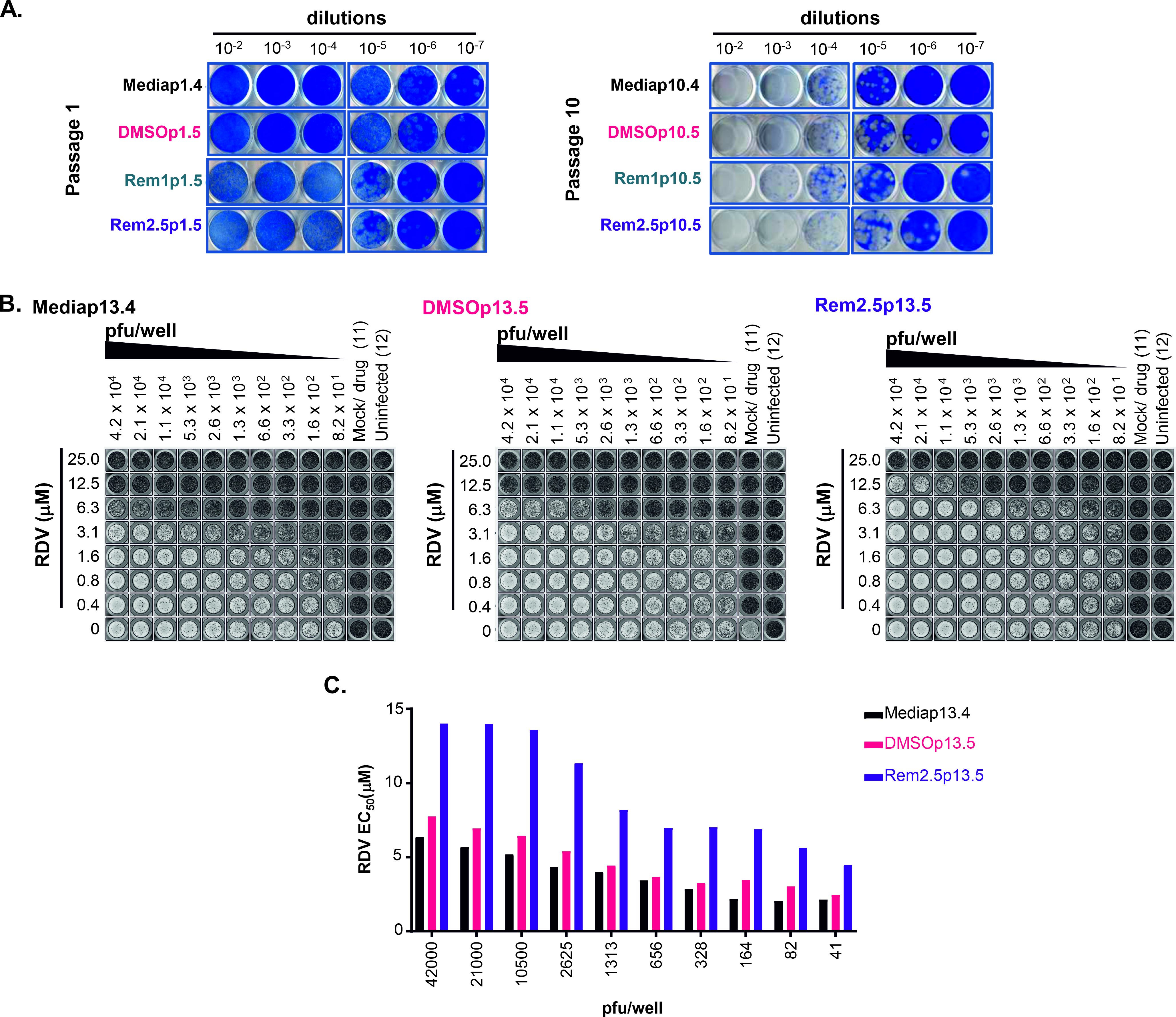

Supplement: S2 Fig — (A) Coomassie blue stained SARS-CoV-2 plaque assays in VeroE6. Change in plaque morphology from passage 1 and passage 10. A subset of populations from the 4 different conditions were compared. (B) Celigo scans of Coomassie blue stained 96-well format mixed array drug for two control populations (Mediap13.4 and DMSOp13.5) and a RDV adapted population (Rem2.5p13.5). RDV concentration and virus input is indicated. (C) Bar graph of RDV EC50 for an RDV adapted population and two control populations over a range of virus inputs. (TIF) [file ppat.1009929.s002.tif]

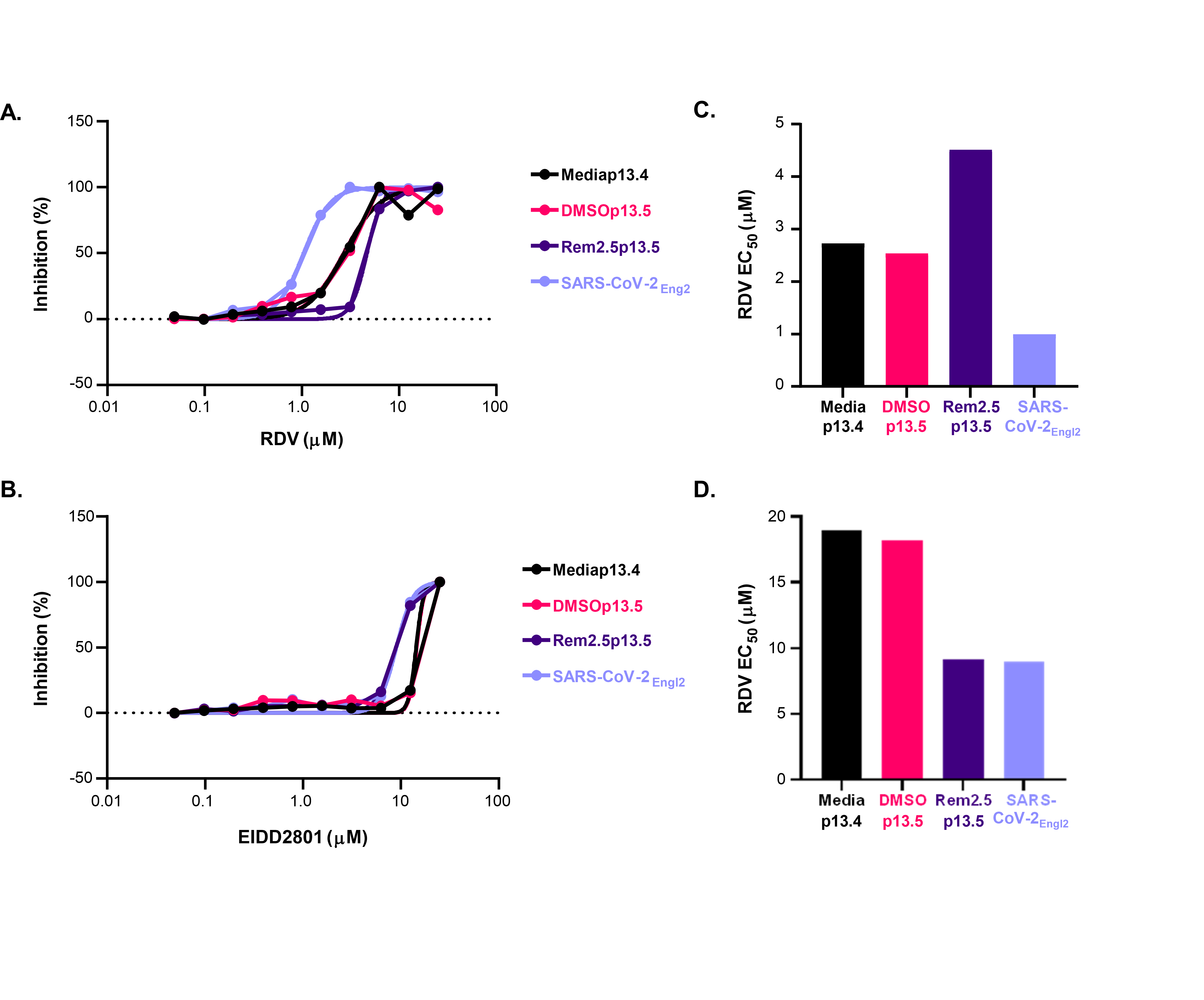

Supplement: S3 Fig — The EC50 of RDV adapted population REM2.5p13.5 was compared to the input virus SARS-Cov-2Engl2 as well as DMSOp13.4 and Mediap13.4. (A) RDV dose dependency curve. (B) Bar graph of RDV EC50 required to protect the monolayer. (C) EIDD2801 dose dependency curve. (D) Bar graph of EIDD2801 EC50 required to protect the monolayer. (TIF) [file ppat.1009929.s003.tif]

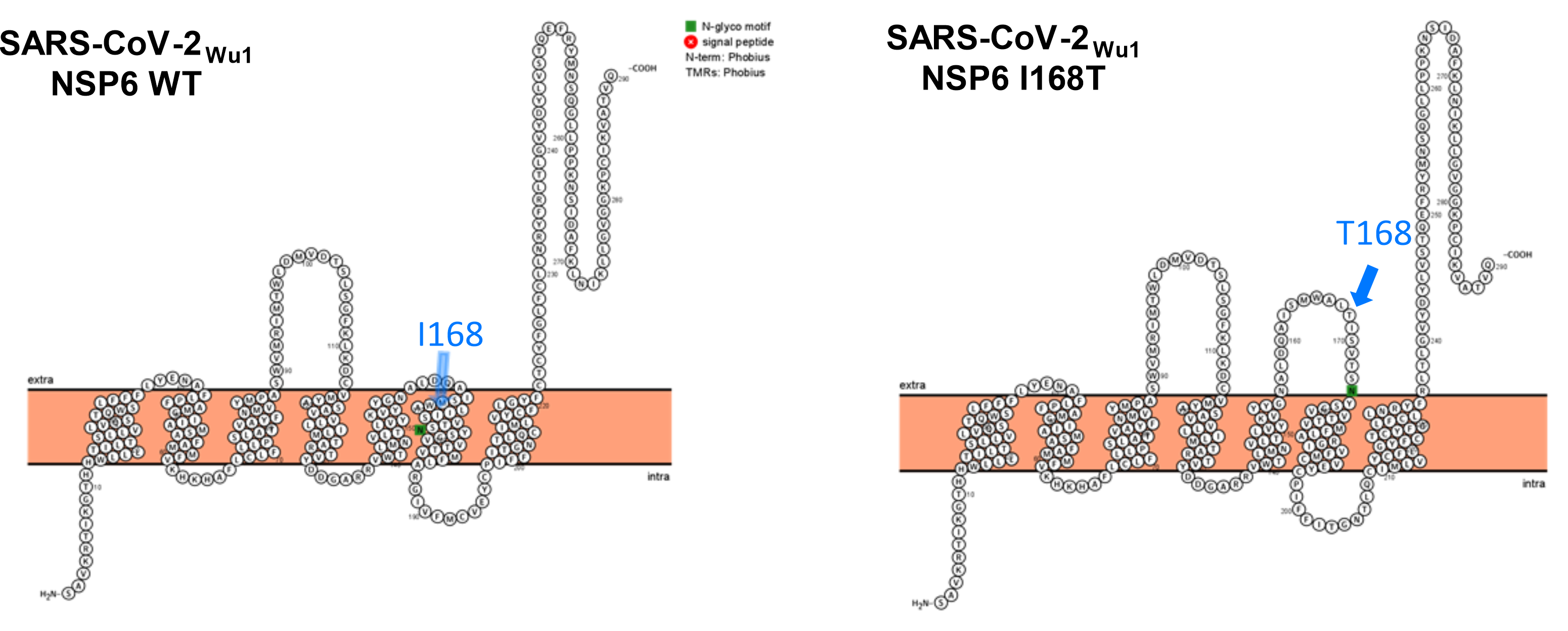

Supplement: S4 Fig — Protter prediction transmembrane organisation of NSP6 WT (left) and NSP6 I168T (right) mutation. Position of I168 and I168T are indicated by an arrow. Extra-, intra-cellular and membrane are indicated, and putative glycosylation site is shown in green. (TIF) [file ppat.1009929.s004.tif]

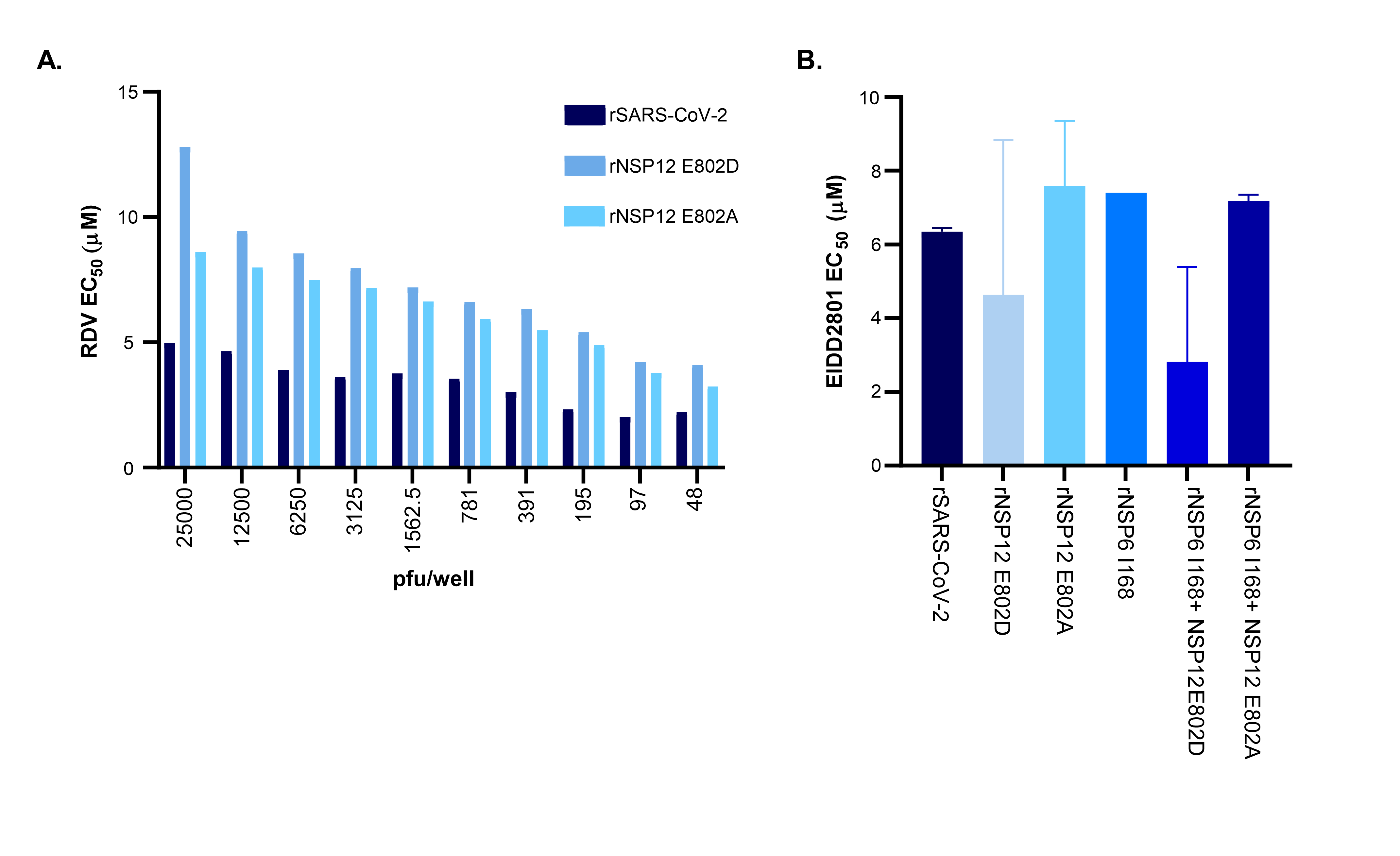

Supplement: S5 Fig — All viruses have a SARS-CoV-2Wu1 backbone with specific point mutations as indicated. Assay undertaken in VeroE6-ACE2-TMPRSS2. (A) Bar graph of RDV EC50 values for rNSP12E902A, rNSP12E802D and rSARS-CoV-2 over a range of virus inputs. (B) Bar graph of EIDD2801 EC50 for each rescued virus. Error bars are SEM. Experiment from 2 independent virus stocks with 3 technical replicates. (TIF) [file ppat.1009929.s005.tif]

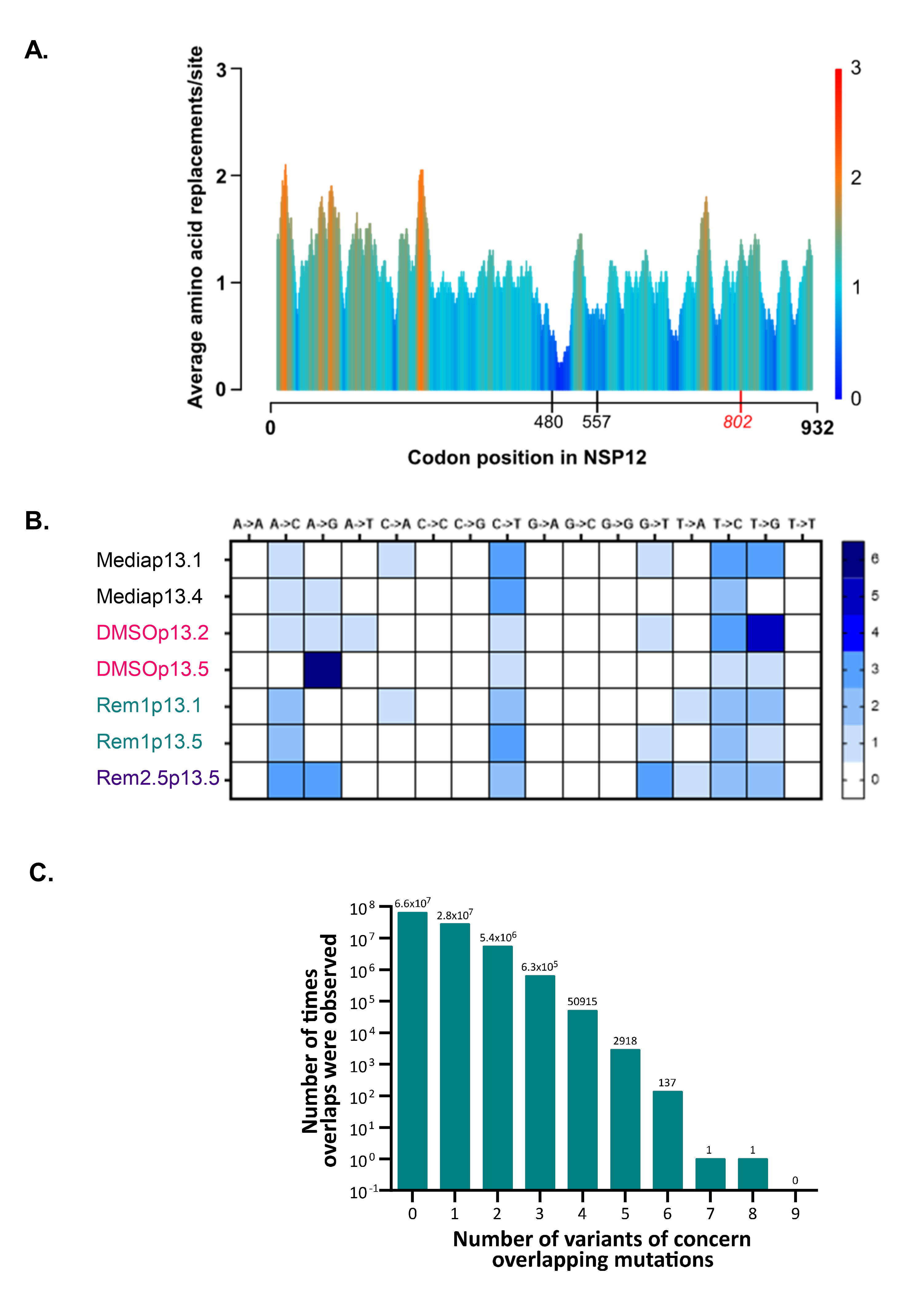

Supplement: S6 Fig — (A) Diversity occurring in SARS-CoV-2 NSP-12 in 20 amino acid sliding window. Calculated from 988 mutations observed in minimum of 5 sequences. Position of mutation associated with decrease RDV sensitivity in SARS-CoV-2 is highlighted in red and sites identified in murine hepatitis virus (MHV) and Ebola virus (EBOV) are indicated in black. (B) Heatmap of the distribution of transversion and transition depending on nucleotide. (C) Distribution of convergence. The null distribution of overlapping in vitro mutations hitting the same Spike codon positions as in the SARS-CoV-2 variants of concern. 21 in vitro mutations and 20 variant of concern mutations spread across 1271 codons. 100 million simulations were run. (TIF) [file ppat.1009929.s006.tif]

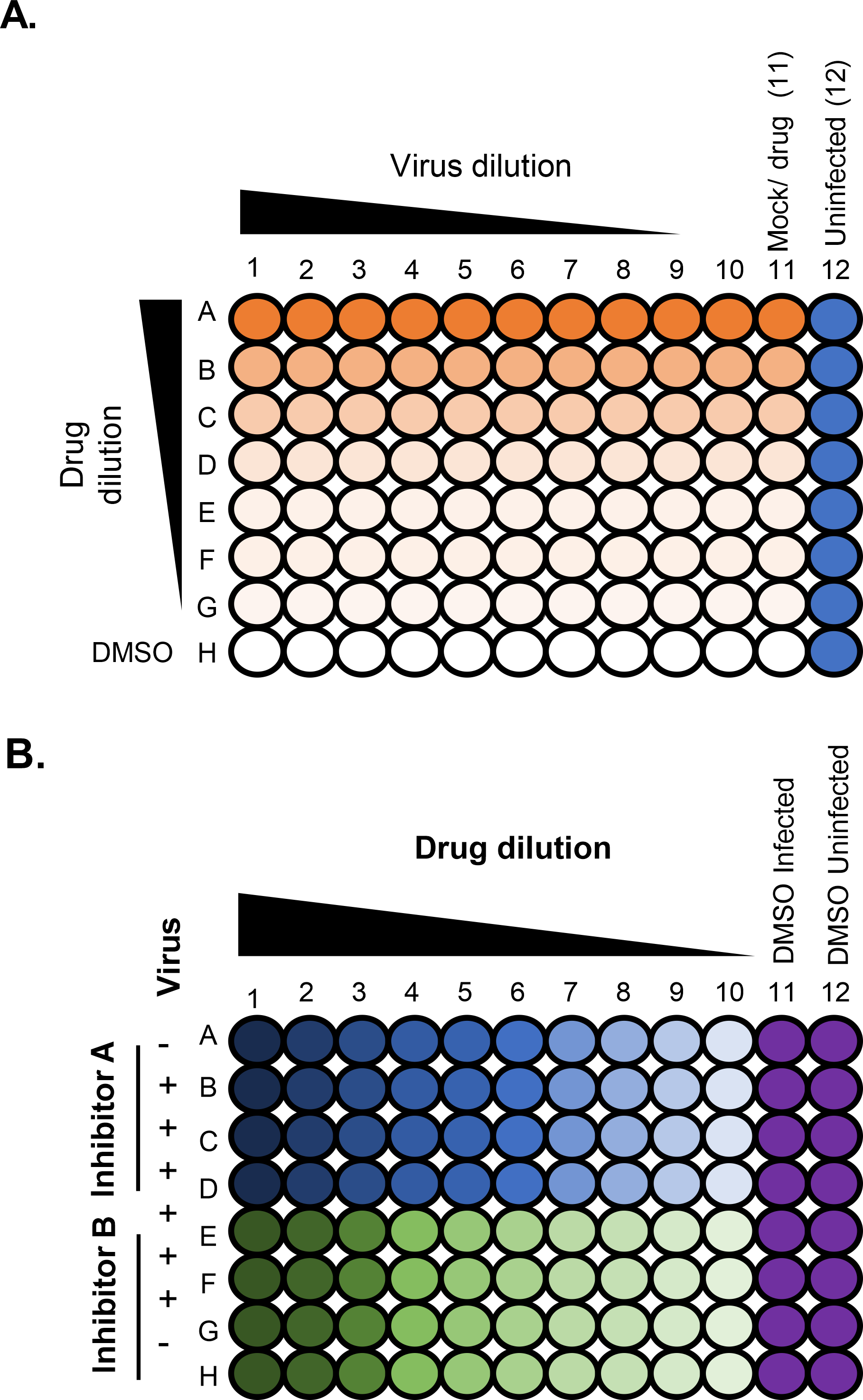

Supplement: S7 Fig — All layouts have controls for inhibitor toxicity, virus clearance and cell monolayer integrity (DMSO only). (A) Mixed array layout with range of inhibitor dilutions vs a range of virus dilutions. (B) Mixed plate layout inhibitor is diluted and a set amount of virus. Amount of virus added causes complete well clearance by 72 h pi. The mixed plate layout can also be modified to dilute inhibitor down the rows rather than across the columns. (TIF) [file ppat.1009929.s007.tif]
