# Supplementary material for: In vitro selection of Remdesivir resistance suggests evolutionary predictability of SARS-CoV-2
Source: PLoS Pathog. 2021 Sep 17;17(9):e1009929. doi: 10.1371/journal.ppat.1009929 (PMC8496873; doi:10.1371/journal.ppat.1009929)
Supplement: S1 Text — (DOCX) [file ppat.1009929.s014.docx]

# Supporting Text

*Adaptation of SARS-CoV-2 to VeroE6*

General adaption of the virus led to an over shift in the EC_50_ of the continually passaged virus in comparison to the input virus SARS-COV-2_Engl2_. This was clear with both the RDV EC_50_ and EIDD2801 EC_50_ for DMSOp13.5, and Mediap13.4 virus lineages (S3 Fig) determined in cell lines with a VeroE6 backbone. This was a 2.5 to 2.7-fold and 2.1-fold increase in RDV and EIDD2801 EC_50_ required to protect the cells against a dose of SARS-CoV-2_Engl2_ (S3 Fig) that would cause complete clearance of a 96-well. The RDV EC_50_ fold-change for the REM2.5p13.5 in these cells was 4.54 when compared to SARS-CoV-2_Engl2_ and was 1.64 to 1.78-fold higher when compared to the continually passaged viruses. This can be directly compared to the fold change of the RDV EC50 for the same viruses in A549NPro-ACE2 cells. The fold-change of viruses passaged in the absence of RDV was equivalent to SARS-CoV-2_Engl2._ DMSO adapted viruses were more sensitive to RDV while the media adapted viruses were similar to SARS-CoV-2_Engl2_. There are other caveats that should be consider including metabolism of the antivirals to their active forms between the two cell types [1,2]

*Does RDV cause SARS-CoV-2 to mutate?*

It is not an uncommon phenomena that nucleoside analogue cause higher mutation rates in RNA viruses [3,4]. While there is no difference in the number of mutations that accumulated between the virus passaged in low concentration of RDV treated samples (Rem1p13.1 and Rem1p13.5) and the virus population grown in the absence RDV (Fig 4 and S1 Data). There is, however, a very clear difference in number of mutations accumulated Rem2.5p13.5 in comparison to all other passaged viruses. This may infer that higher concentration of RDV does increase mutation rate. We would suggest caution is used when making this conclusion as sample size for all groups was small and we have no replicates for viruses adapted in a higher concentration of RDV.

*Are the samples under selective pressure to evolve?*

Calculation of the dN/dS ratios of the concatenated genomes showed clear evidence of positive selective pressure in a majority of the continually passaged viruses regardless of the presence of RDV (Table B). This is further supported when examining Spike and ORF1ab individually. As mentioned, this needs to be caveated with our small sample size, the high dN/dS ratios are influenced by the lack of observed syn mutations. (i.e., if 0 syn mutations and >0 non-syn mutation, then dN/dS will be infinity).

*Are the sites in Spike under adaptive pressure to change?*

We were surprised to observe the mutation of Spike in our passaged viruses that were occurring in the same location as those identified in the newly emerging variants from the UK, South Africa and Brazil that are associated with increased transmission [5]. The current hypothesis suggests that these mutations are due to immune selection and/or pressure. Our data would suggest that SARS-CoV-2 can and does use positions to allow adaptation to very different conditions. These Spike mutations arose and fixed in our passaged viruses; I68R, H69R, T95I, N211K, E484D, N501T, I569S, Q613R, I624V, H655Y, P681P, S708N/F/F, N709H, T723A, P728P, V729V, D985G, V1128F, and G1219C. Not all mutations occurred in every lineage, and the frequency also varied between lineages. There were also 14 positions with specific substitution arising at a frequency of 5 to 30%, this included two sites in the RBD: G413R and Q498H.

We are confident selective pressure occurring with substitution of E484 (10% to 80% fixed) and H655 (46 to 99% fixed in 5 populations) arising within all 7 passaged. While N501 mutation was in 2 lineages (16 to 61%). There are also synonymous changes occurring at both P681 and P728 rather than non-synonymous change associated with the circulating variants. Furthermore, we have a substitution at H69 rather than a deletion. The T95I and P681 changes were present in the input virus but otherwise the others have arisen through passage. A previous publication [6] highlighted that the *in vitro* mutation rate of SARS-CoV in VeroE6 was negligible indicating that it was well-adapted for growth in cell culture. This would indicate that there is further selective pressure on these sites in Spike to change in independently evolving virus populations to provide an advantage.

To further examine the likelihood of the observed overrepresentation of the 21 variant of concern defining spike mutations in our *in vitro* passaged viruses, we simulated mutational events under a null distribution. This null distribution assumed that all codons were equally likely to be mutated within Spike (except for the start and stop codons; amino acid positions 1 and 1273 respectively). Each run involved 20 codons in Spike being sampled (without replacement- i.e., each codon could be mutated maximally once), and the number of sampled codons which overlapped the VOC sites were recorded. The distribution (S6 Fig) over 100 million simulations only produced five overlapping mutations 2981 times, giving a probability of 3.1x10^-5^ observing this level of overlapping evolution if the *in vitro* evolution was random. It is important to note that the null distribution was not impacted by the single deletions of amino acids 68 and 69 in the UK B.1.1.7 variant as mutations of these sites in the *in vitro* sequences required two mutations in the *in vitro* data.

We further investigated the global distribution of the mutations within in Spike to examine whether our *in vitro* substitutions occurred within hot spots of amino acid replacements in the global SARS-CoV-2 sequence database. In Fig 4E, each bar on the y axis represents a sliding window of 20 amino acids, counting the average number of unique amino acid replacements observed for each site in that window. The method we used does not incorporate information from the phylogeny and only counted unique observed states, as recorded in CoV-GLUE, using GISAID data up to the 14^th^ of December 2020. For example, the N501Y replacement has occurred numerous times in parallel (<http://cov-glue.cvr.gla.ac.uk/#/project/replacement/S:N:501:Y>), but it will only be counted once in our analysis, though the N501T and N501S substitutions will be counted as additional replacement (<http://cov-glue.cvr.gla.ac.uk/#/project/replacement/S:N:501:T> ; <http://cov-glue.cvr.gla.ac.uk/#/project/replacement/S:N:501:S>) In order to avoid counting sequencing errors or strongly deleterious variants, amino acid replacements seen in fewer than five independent sequences on the CoV-Glu database were ignored (for tree aware selection analyses, which incorporate observations of parallel mutation events, see <https://observablehq.com/@spond/revised-sars-cov-2-analytics-page>). Figure 4E shows clear overlap and shared clusters of substitution shared between variants of concern and the *in vitro* passaged virus within Spike.

It should be noted that reference stock of SARS-CoV-2_Engl2_ provided from PHE underwent two rounds of amplification in VeroE6 prior to use in our continual passage study (Fig 1A). Deep sequence of the amplified stock revealed diversification of the amplified virus away from the reference sequence. There were five derived non-reference mutations present at a frequency of ~50%, four of these five became fixed across all passaged populations by p13. (S1 Data). In our analysis of substitutions of Spike, we have included T95I as a tissue culture derived mutation as it was at ~46% frequency in the input virus and fixed at 99 to 100 in all bar one virus (Fig 4A).

**Supporting Reference**

1. Pruijssers AJ, George AS, Schäfer A, Leist SR, Gralinksi LE, Dinnon KH, et al. Remdesivir Inhibits SARS-CoV-2 in Human Lung Cells and Chimeric SARS-CoV Expressing the SARS-CoV-2 RNA Polymerase in Mice. Cell Rep. 2020;32: 107940. doi:10.1016/j.celrep.2020.107940

2. Mumtaz N, Jimmerson LC, Bushman LR, Kiser JJ, Aron G, Reusken CBEM, et al. Cell-line dependent antiviral activity of sofosbuvir against Zika virus. Antiviral Res. 2017;146: 161–163. doi:10.1016/j.antiviral.2017.09.004

3. Graci JD, Cameron CE. Challenges for the Development of Ribonucleoside Analogues as Inducers of Error Catastrophe. Antivir Chem Chemother. 2004;15: 1–13. doi:10.1177/095632020401500101

4. Fung J, Lai C-L, Seto W-K, Yuen M-F. Nucleoside/nucleotide analogues in the treatment of chronic hepatitis B. J Antimicrob Chemother. 2011;66: 2715–2725. doi:10.1093/jac/dkr388

5. Galloway SE, Paul P, MacCannell DR, Johansson MA, Brooks JT, MacNeil A, et al. Emergence of SARS-CoV-2 B.1.1.7 Lineage - United States, December 29, 2020-January 12, 2021. MMWR Morb Mortal Wkly Rep. 2021;70: 95–99. doi:10.15585/mmwr.mm7003e2

6. Vega VB, Ruan Y, Liu J, Lee WH, Wei CL, Se-Thoe SY, et al. Mutational dynamics of the SARS coronavirus in cell culture and human populations isolated in 2003. BMC Infect Dis. 2004;4: 32. doi:10.1186/1471-2334-4-32
