# Supplementary material for: In vitro selection of Remdesivir resistance suggests evolutionary predictability of SARS-CoV-2
Source: PLoS Pathog. 2021 Sep 17;17(9):e1009929. doi: 10.1371/journal.ppat.1009929 (PMC8496873; doi:10.1371/journal.ppat.1009929)
Supplement: Attachment — Submitted filename: Szemiel_RDV_rebuttal_final.docx [file ppat.1009929.s015.docx]

We thank the reviewers for helpful comments and suggestions. We hope to have addressed all points in a comprehensive manner.

**Reviewer #1:**

***1. Many studies have shown multiple spontaneous mutations on spike protein when serial passage of SARS-CoV-2 on Vero-E6 cell line are under selective pressure (Ex. Sasaki M, Uemura K, Sato A, Toba S, Sanaki T, Maenaka K, et al. (2021) SARS-CoV-2 variants with mutations at the S1/S2 cleavage site are generated in vitro during propagation in TMPRSS2-deficient cells. PLoS Pathog 17(1):e1009233. Or Journal of General Virology 2020;101:925–940) These studies suggested that virus variants with spike gene mutations generated smaller plaques as shown in this study due to the loss of the ability to utilize the entry pathway involving TMPRSS2 and alerted the researchers propagating the virus in tissue culture without TMPRSS2. Therefore, the mutations on spike protein gene generated through serial passage of SARS-CoV-2 on vero-E6 cell might be due to (1) spontaneous mutations irrelevant to RDV, or (2) concordant mutations due to RNA secondary structure upon RDV treatment, or (3) change in receptor usage upon RDV treatment since the lack of ACE2 receptor on vero-E6, which may allow the virus to use other method of entry. Further experimental confirmation or detail discussions is demanded.***

The key goal of the study was to identify mutations conferring resistance to remdesivir. In Figure 4, we provide the reader with an overview of all the mutations arising in the passaged viruses in the presence or absence of remdesivir. We agree with the reviewer that mutations and deletions can and do arise in cell-culture based passage, including the loss of the multi-basic cleavage site observed in some studies as mentioned by the reviewer. In our study however, we simply highlight that some of the amino acid mutations that arise thorough passage *in vitro* are also key residues mutated in some variants of concern. The key message that we provide is the selective pressure on these mutated residues is likely not due to immune-escape mechanisms.

We appreciate that other papers have specifically focused on mutations in VeroE6 (that we have now cited accordingly) including those that have observed loss of multi-basic cleavage site as well those that have not (Rihn et al., 2020, doi: 10.1371/journal.pbio.300109; Moreno et al., 2020, doi: 10.1128/JVI.02209-20; and Chiem et al., 2021, doi:10.1128/JVI.02209-20). Furthermore, since the submission of this paper, other studies are now identifying some of the same mutations associated with variants of concern in Spike during in vitro passage (Raminez et al., 2021 doi:10.1128/AAC.00097-21; Braun et al., doi:10.1371/journal.ppat.10093730)

We have addressed the 3 main points highlighted by the reviewer separately.

*(1) Spontaneous mutations irrelevant to RDV*

Changes in the viral genome during cell passage are indeed to be expected and could be due to a variety of reasons related to the environment provided by VeroE6 cells. However, the key point that we thought was important to stress in our data were the spike mutations we observed *in vitro*. The mutations we did observed were predominantly located in the Spike (strong positive selection in this region) rather than arising spontaneously throughout the genome, and often appeared and fixed in more than one lineage (ie., E484 and H655). They also occurred in hot spots of diversity observed in circulating strains.

We have also expanded and clarified the sections relating to them discussion (see lines 337-341) *‘**The high ratio of non-synonymous to synonymous mutations we observed in Spike (Table.S2) was driven by positive selection and not neutral evolution. Recently published studies have also shown the emergence of mutations in SARS-CoV-2 Spike during in vitro passage at the same residues we identified including E484 and H655 [53,54].’*

*(2) Concordant mutations due to RNA secondary structure upon RDV treatment*

To date none of the studies investigating RDV resistance or use as a viral inhibitor have reported changes in RNA structure (Agositini et al., 2018, doi.org /10.1128/mBio.00221-18 and Lo et al., 2020, doi:10.1073/pnas.2012294117). The action of RDV is via template stalling (Kokic et al., 2021, doi:10.1038/s41467-020-20542-0) and it has not been shown to act as a mutagen like other nucleoside analogues including like ribavirin and EIDD2801 (Grotty et al., 2000 (doi:10.1038/82191) and Gordon et al., 2021,doi:1016/j.jbc.2021.100770)

See Discussion lines 316-324, stating *‘…Coronaviruses correct most errors that occur during genome replication. Therefore, if mutations arise and are fixed there must be a general benefit that could include inhibitor escape……… whereas the template stalling action of the RDV limits the spontaneous mutations and therefore the changes we observed in vitro are an evolutionary fine tuning of the viral proteins to a new host…’*

Furthermore, If RDV was driving specific mutation due to compensatory changes in the RNA structure, we would have expected to have observed more sites in common arising between the three populations grown in RDV. Indeed, we only observed changes to 3 sites that occurred in at least two of the RDV passaged population, and none of these occurred in all three populations. That suggests that we can rule out this specific possibility.

*(3) Change in receptor usage upon RDV treatment since the lack of ACE2 receptor on vero-E6, which may allow the virus to use other method of entry*

There is exogenous expression of ACE-2 in the VeroE6 used in this study, these cells were characterised prior to use (see Rihn et al., 2020, doi: 10.1371/journal.pbio.3001091 for the data). Thus, it is unlikely that an ACE-2 deficiency is driving the mutations within Spike in our virus populations.

As is rightly pointed out, SARS-CoV-2 can use multiple pathways to enter cells and, recently, while TMPRSS2 plays a role in determining the entry pathway it is able to use Cathepsin L/B as an alternative protease (Koch et al., 2021.doi: 10.15252/embj.2021107821; Peacock et al., 2021, doi:.1038/s41564-021-00908-w; Hoffman et al., 2020, doi:10.1016/j.cell.2020.02.052).

Nonetheless the point is relevant, and we have expanded on this in the Discussion lines 360-365 *‘The pathways used for entry by coronavirus is dependent on either TMPRSS2 or Cathepsin L expression [63–65]. VeroE6 are generally TMPRSS-2 deficient but over express and process Procathespsin L to the active cathepsin L in abundance [64]. We postulate that this over expression of Cathepsin L allows for the maintenance of the multi-basic cleavage site in our continually passaged SARS-CoV-2 lineages…*’

Unlike the studies mentioned (Sasaki et al., 2021, doi: 10.1371/journal.ppat.1009233; Klimstra et al., 2020, doi: 10.1099/jgv.0.001481 and Davidson et al., 2020, doi:10.1186/s13073-020-00763-0) we did not observe any mutations/deletions in the multibasic cleavage site. There are also other studies where the deletion is not observed in the reverse genetic derived SARS-CoV-2 after multiple passages (Rihn et al., 2020, doi: 10.1371/journal.pbio.300109; Moreno et al., 2020, doi: 10.1128/JVI.02209-20; and Chiem et al., 2021, doi:10.1128/JVI.02209-20).

Again, we have expanded on this point in the Discussion see lines 357-360 onwards.

*‘Unlike other studies using VeroE6 [25,26], we did not observe the rapid loss of the multi-basic (furin cleavage) site in SARS-CoV-2 spike in vitro. The cleavage of spike into the S1/S2 subunit is critical for virus entry and can be processed by multiple proteases including furin, serine protease (TMPRSS2/4) and the cysteine proteases Cathepsin B/L [61–63].*

And lines 365-366 ‘*Other studies have also shown that the proteolytic cleavage site is maintained in vitro continual passage for SARS-COV-2 [66–68].’*

**2. Certain mutant virus arises under selection pressure with the cost of fitness lost. Such mutant virus lost the growth under competition with the wild type virus. The authors should investigate any fitness change by mixing the mutant virus with the parental virus with different ratio.**

We thank the reviewer for the useful suggestion. We have undertaken an *in vitro* based competition assay mixing wt and NSP12 E802D mutant at either a 1:9 or 9:1 ratio. The data clearly demonstrated that the NSP12 E802D mutation affected virus fitness.

The information pertaining to the results, methods and discussion can be found from lines 167-181 onward in the Results *‘**To examine whether the viruses containing the NSP12 E802D mutation would persist and out compete the WT in the absence of RDV, an in vitro co-infection competition assay over multiple passages was undertaken………. indicate the E802D mutation incurs a fitness cost in the absence of RDV and is unlikely to be sustained in the circulating population.’*

lines 285-290 in the Discussion *‘**The in vitro mutations associated with partial RDV resistance for SARS-CoV-2 as well as those identified in MHV and SARS-CoV-1, circulate at extremely low frequency.……… EBOV that conferred partial resistance and where the frequency was maintained in competition assays [21].*

lines 483-510 onwards in the Methods and Materials under the following new sections ‘*Co-infection competition assay Quantification of SARS-CoV-2 genome copies. … Sequence of virus stock using GridION (Oxford Nanopore Technology*).

Figure 3 now has two extra panels illustrating the data from the competition assay (Fig.3D and Fig.3E).

**3. Figure S1 should illustrate what the difference is between mock and 0 pfu/well. And why the input virus (1024-32pfu/well) at left and (14-1pfu/well) at right showed the lightest colour representing clearance of monolayer, compared to high input viruses such as 32768pfu/ml.**

We apologise if this was not clear and have improved the figure. The pfu/well are the titres based on either a 2-fold or 3-fold dilution. Both the 2-fold and 3-fold had the same starting input. To avoid confusion, we changed 0 pfu to 0.5 pfu in Figure S1. We have now included the word uninfected in association with the mock in Figure S1.

During the initial development of the assays, we observed that that high MOI infection with SARS-CoV-2_Engl2_ of VeroE6 did not result in well clearance in a TCID_50_/ml (see below). However, as the virus adapted to the cells, well clearance was observed. Therefore, when the intensity was determined for the well as shown in the Figure S1, at high virus input the intensity is similar to uninfected cells in colour, whereas lower virus input had less intensity and therefore a lighter blue.

**SARS-COV-2 passaged in Media**

Passage 1

Passage 7

**10-fold serial dilution**

**4. Line 109-110, could the authors explain why Rem1p13.5 was selected for EC_50_ testing instead of testing all lineages which Rem1p13 survived?**

We did undertake an initial preliminary screen with 5µM RDV and 5-fold dilutions of all 24 independently generated lineages including the 6 populations from Rem1 using passage 12, to reduce the number of screens and reagents that would be subsequently required. We did not include this data in the paper.

**5. From Figure 4, it is not clear if the authors sequenced all the lineages of survived viruses as mentioned in Line 116 by the authors. Since multiple spontaneous mutations could occur when serial passage of SARS-CoV-2 on Vero-E6 cell line, sequencing all the lineages could assure the results illustrated in section “Common mutations in two virus populations with partial resistance to RDV”. It is important since natural resistant viruses can occur under just DMSO treatment as shown in Fig 1C. If the authors did sequence all the lineages of viruses, a consensus map like figure 4 showing all the sequenced viruses will be better than just providing datafileS1.**

We have clarified that we did not sequence all viruses from all lineages by stating we sequenced a subset of population (see lines 115 ‘…*the consensus sequences from a subset of viruses passaged..*’).

We sequenced 2 virus populations from each condition, those from the untreated and DMSO lineages were randomly selected. As mentioned above, we had undertaken a preliminary screen to identify virus populations that were able to cause well-clearance in 5µM RDV with at least 1 log_10_ less virus than those that were grown in the absence of RDV, thus reducing the number of viruses we needed to sequence.

The consensus map of the fixed mutations for all the sequenced lineages are shown in Fig.4.

We also clarified that the analysis is from a subset rather than all lineages in the section *in vitro* mutations provide insight into Spike adaption line 213 onward *‘….of a subset of the passaged stocks….’*

**6. Line 144-147, need to specify what kind of statistics was used to get p value and p-adj? And what p-adj mean? Meanwhile, Line 406-408, need to specify which statistics was used in which experiments?**

We have specified the statistics used in the Methods line (line 517 ‘…*RM one-way ANOVA (Dunn Dunnett's multiple comparisons test), ….’*) and in the Results section (line 147 onward *‘…RM One-Way Anova with Dunnett’s Test…*’), where necessary.

The p.adj values are from using the Dunnett’s test within the RM One-Way Anova within the Prism package. The p.adj is more stringent than the p value, as it considers the false discovery rate and as with p for p.adj to be significant p.adj =0.05. We also undertook two-way ANOVA and student-test comparisons to ensure that there was no significant difference between the wt and mutant viruses at any of the time points.

Further, we used the opportunity to correct the text from p = to p.adj = rSARS-CoV-2 vs rNSP12-E802A (line 146)

**7. Figure 3C, mutant virus rNSP12-E802D showed decrease in titer from 10^2 at 24hr to 10^1 at 48hr under 1uM of remdesivir. However, the same mutant virus showed constant in titer of 10^1.5 at 24hr and 48hr under 2uM of remdesivir. Could the authors explain why the mutant virus grows inferior under low concentration of RDV? Could the titer of the mutant virus increase when culture 72 hr under 1uM of RDV**?

As the reviewer points out, at 24 h pi we have a plateau of virus titre for both NSP12 E802D between 10 and 100 pfu/ml in RDV concentration from 0.5 to 2µM, whereas the 48 hr in 1µM RDV does have detectable virus in the 2µM RDV at 48h. It should be noted that one of the NSP12 mutants E802A does have detectable virus at 48 h when grown in 1µM RDV. The elevation of RDV restriction by E802 is only partial, similar to the MHV mutants. Not mutation that provides full resistance to RDV has been identified.

There are possible explanations for this, and we have discussed how SARS-CoV-2 is able to overcome RDV restriction in the updated Discussion (Lines 296-298)

*‘… The interplay between balance of naturally occurring dinucleotide concentration [3,36] and the cell’s ability to metabolize RDV to its active form [4] are likely to influence whether break-through replication will occur*.’

Pruijsser et al., (2020 doi: 10.1016/j.celrep.2020.107940) showed different cell types have different rates of RDV-TP formation, thus depending on cell type at earlier timepoints ie., 24 h at higher concentration of RDV not enough metabolised RDV-TP is present, therefore the EC_50_ are different for different cell types. Secondly, Gordon et al., (2020, doi: 10.1074/jbc.RA120.013679) showed that the RNA stalling caused by RDV incorporation can be overcome with higher concentrations of natural nucleotide pools. Therefore, we could hypothesise that after 24 h the incorporation of RDV-TP into the genome was not sufficient to prevent the stalling of the polymerase, though the second round of infection may have resulted in the incorporation of more RDV molecules into the genome.

We only examined the titres at 24 h and 48 h as it is equivalent to first two rounds of replication and after 48 h the virus is in a plateau phase based on our growth curves in Calu-3 (Fig.3A). Thus, whether the titres of the mutant viruses in 1µM would increase at 72 h would be speculative. The titre would increase depending on whether there was sufficient RDV-TP to be incorporated into the genome and whether the intracellular pool of dinucleotides remained sufficient to overcome any restriction. Pruijsser et al., (2020 doi: 10.1016/j.celrep.2020.107940) showed interesting research in this regard, by using SARS-CoV-2 2019-nCoV/USA-WA1/2020 clinical isolate in a subclone of Calu-3 (Calu3 2B4 cells) showed higher titres at 72 h in 1µM RDV, so indeed it is possible. Whether the titres of the mutant virus (rNSP12E802D) in 1µM would increase at 72 h would be speculative, as the rNSP12E802A was detectable indicates that it is indeed possible.

**Reviewer #2**

**In vitro Remdesivir resistance was acquired rapidly. The mutations described here are not frequent in SARS-CoV-2 cases. They also do not emerge in documented long-term infections of patients who underwent Remdesivir treatment. The authors should discuss this disconnect.**

We have expanded the Discussion to address these points see lines 270 onwards ‘…*with at least one lineage appearing within 4 passages (Rem2.5 lineage*) ….

We observed breakthrough at passage 4, but did not confirm resistance until passage 10, when we undertook a mini screen that enable us to focus on those preparations that had potential resistances. With Ebola RDV resistance, the mutation that conferred resistance was first observed in the sequence at passage 9 but not fixed until much later passages (passage 23; Lo et al., 2020, doi: 10.1073/pnas.2012294117).

The competition assays we carried out demonstrated that these remdesivir-resistant mutant have a fitness cost, and this explains why there is an extremely low frequency of this mutation in naturally circulating strains. We have expanded on the fitness of RDV mutation and the reason why they have not increased in the population in the Discussion (line 285-292)

*‘**The in vitro mutations associated with partial RDV resistance for SARS-CoV-2 as well as those identified in MHV and SARS-CoV-1, circulate at extremely low frequency. This could in part be attributed to the a decrease in fitness of coronaviruses with these RdRp mutations, as observed in competition assays results [20,21]. Indeed, we found that SARS-CoV-2 NSP12 E802D were outcompeted by wild type virus in our in vitro assays. This is unlike RdRp mutations in EBOV that conferred partial resistance and where the frequency was maintained in competition assays [21].’*

This was also addressed by a competition assay with two different ratios of rNSP12-E802D for SARS-CoV-2 which showed that the wild type out-competed the mutant. The information regarding the competition assay is found on lines 167-181 (Results ‘*To examine whether the viruses containing the NSP12 E802D mutation would persist and out compete the WT in the absence of RDV…),* lines 285-290 (Discussion ‘*The in vitro mutations associated with partial RDV resistance …’)* and lines 483-510 onwards in the Methods and Materials starting ‘*Co-infection competition assay…’*).. There are also two extra panels in Fig.3 with the associated results.

There are only a handful of papers with patients with long-term RDV use, we have included a few of these within our Discussion (lines 276-284)

*‘….**In a clinical setting, there has not been any evidence of RDV resistance arising in patients. Two studies showed clearance of viraemia after the second treatment but not after the first [29,30]. In the study reported by Kemp et al., [31] …………… the 10-day treatment RDV regime for the clearance of SARS-CoV-2 and the development of resistance may requires longer treatment periods in patients before this phenotype becomes noticeable…’*

In these studies, patients are generally immuno-compromised and/or have underlying conditions. Helleberg et al., (2021, doi: 10.1093/infdis/jiaa446) and Buckland et al., (2021, doi: 10.1093/infdis/jiaa446) show clearance of viraemia after the second treatment but not after the first. Whereas, in the study reported by Kemp et al., (2021, doi: 10.1038/s41586-021-03291-y) virus was never cleared or suppressed by any of the treatments. Based on this they speculated that a V157 responsible for the resistance. We and others have not seen this mutation either *in vitro* or *in vivo* experiments.

Perhaps, the development of resistance requires much longer treatment periods (greater than 10 days) than was carried out in patients before resistance becomes noticeable.

**The discussion of the spike mutations requires more nuance. The acquired mutations that confer antibody resistance are at sites that tolerate mutation. Selection by antibodies can result in selection for variants at these positions. The authors have identified sites that can mutate when viruses are passaged extensively. Most discussion surrounding substitutions at 501 and 655 has not been centered on antigenicity.**

We appreciate this comment and have expanded in the Discussion on the topic of mutations within the Spike (lines 343-356), *‘…**This is further highlighted by two mutation that arise in vitro; N501T and H655Y, the first within the RDB and the latter outside of the RDB and toward the S1-S2 cleavage site. Mutation of N501 increased affinity to human ACE-2 [43,45] ……………… While not in the scope of this paper, functional studies are recommended. While not in the scope of this paper, functional studies related to change observed during in vitro evolution experiments can help to identify candidate amino acid residues likely to drive future viral adaptation.*

This includes that N501 is a site that is also under selection for adaptation from civet to human ACE-2 for SARS-CoV-2. We have introduced a point into the Discussion regarding substitutions of H655 based on the currently literature, although as highlighted by Braun et al., (2021, 10.1371/journal.ppat.1009373) the phenotypic role of this mutation is still unknown - even though it evolves in several species and has arisen in independent lineages. It is postulated to be involved in stabilising the cleavage of spike to S1/S2. Indeed, in our data the only passaged virus (DMSOp13.2) that contained deletion of the multi-basic cleavage site, did not have the H655 substitution.

**Part III – Minor Issues: Editorial and Data Presentation Modifications**
Please use this section for editorial suggestions as well as relatively minor modifications of existing data that would enhance clarity.

**Reviewer #1:**

**Line 105-108, EC50 needs to be consistent since IC50 was used in the figure legend.**

This oversight in the supplementary text has been corrected throughout. We calculated EC_50_ values.

**Line 127, should be “result in” instead of “reduce in”**

We have clarified the sentence by stating the following on lines 125-127

*‘We propose that the E802D mutation changes the steric interactions between amino acid side chains within this region resulting in minor structural changes,…’*

We propose that the reduction in the size of the side chain removes (reduces) the steric clashes occur within this region of NSP12, there are several ‘bulky’ amino acids in this region and may influence have binding of C813 and/or S814 to RDV that will allow stalling and termination to occur and increase the size of the ‘hole’ the RNA template is moving through.

**The naming system should be consistent, such as using SARS-CoV-2wu1 in Line 120 but using rSARS-CoV-2 in line 143 or 151….**

We apologise for the confusion. On line 119, we are referring to the first published sequence of SARS-CoV-2 Wuhan 1 (SARS-CoV-2_Wu1_). We have included the accession number for clarity (line 120 *‘(*[*MN908947*](https://www.ncbi.nlm.nih.gov/gquery/?term=MN908947)*)’*). We used the rSARS-CoV-2 nomenclature to discriminate between the original isolate (SARS-CoV-2_Wu1_) and the reverse genetic derived virus. We have clarified this in sentence starting on line 142 *‘All rescued viruses with specific point mutations replicated similarly to the rescued wild-type virus (rSARS-CoV-2)*’

**Figure 3 should be more self-explanatory by showing the titers tested in which cell lines since 3B was done in vero-ACE2-TEMPRSS and 3C was done in calu-3.**

We have clarified this in the figure legend to include the titres. The legends now read as;

(B) RDV dose-dependent inhibition for each mutant virus. Post-treatment with RDV, VeroE6-ACE2-TMPRSS2 were infected with 500 pfu/ml of each virus. Mutations in NSP12 decrease the sensitivity to RDV (starting line 868).

(C) RDV dose effect on virus titers at 24 h (left) and 48 h (right). Calu-3 were treated with decreasing doses of RDV and infected with an MOI~0.01 (starting line 871).

**Reviewer #2:**

**Unfortunately, since the time of this manuscript’s drafting over a million more deaths due to SARS-CoV-2 have occurred. Before publication, please update this information.**

We have updated this in the text line 51 and added reference to WHO dashboard *‘…caused more than 4 million deaths by July, 2021 [1]…*’. Unfortunately, we believe this will only increase and be outdated soon too.

We also used this opportunity to include the new variant names ie., Alpha, Beta etc in the text were appropriate in line with the new WHO nomenclature for the variants of concern.

**Line 132: Use of highly conserved. Things are either conserved or not and do not need a qualifier.**

This is a fair point. We removed the word highly.

**I found the “Prevalence of RDV mutations in circulating populations” section difficult to follow. Please consider reworking it.**

We have reworded the section, and believe it is now clearer and easier to follow.

Starting line 184 to 194 ‘…We next examined the available SARS-CoV-2 genome sequences in the GISAID database (n=844376 as of March 2021) to determine the frequency of replacements at NSP12 E802 and NSP6 I168. We only identified 36 (0.004%) 4 sequences with E802A substitution from May 2020 from the same geographic region (United Arab Emirates) but there is no evidence of further transmission.
